# A Ten-Year Review of Audiological Performance in Children with Inner Ear Abnormalities after Cochlear Implantation in Singapore

**DOI:** 10.1155/2019/6483714

**Published:** 2019-12-01

**Authors:** Sok Yan Tay, Rosslyn Anicete, Kun Kiaang Henry Tan

**Affiliations:** ^1^Department of Otolaryngology, Head and Neck Surgery, National University Hospital System (NUHS), Singapore; ^2^Department of Otolaryngology, KK Women's and Children's Hospital, Singapore

## Abstract

**Objectives:**

To evaluate children with inner ear malformations following cochlear implantation (CI) in a tertiary pediatric hospital in Singapore to identify factors influencing outcomes after CI.

**Methods:**

This is a retrospective cohort study of children aged 0 to 18 years, who had CI between 2000 and 2013. Demographic information, data on risk factors, type of inner ear malformation (IEM), age at implantation, speech pre- and postimplantation, and duration of follow-up were collected from clinical records. Operative details and audiological outcomes were also analyzed.

**Results:**

A total of 70 children underwent 83 CI surgeries. The mean age of the patients was 4.05 ± 3.17 years (range 1–18 years). Twenty patients (28.57%) had abnormal CT scan findings. CSF gusher occurred in 15 out of 26 CI (57.69%) in the group with IEM. Nine out of twenty patients (45.00%) had poor IT-MAIS scores prior to implantation. The average preoperative IT-MAIS score for children with anomalous inner ear anatomy was 14.1. The older CI patients, 3/20 (15.00%), mean age 8.33 years (range 7–10 years), were mostly referred for persistently unclear speech following hearing aids. Eleven patients (55.00%) had good speech and aided hearing threshold within speech limits after CI and were eligible for reintegration into mainstream schools. Five patients (25.00%) had improvement in speech but continued to receive education in special schools. Four patients (20.00%) had poor progress after surgery.

**Conclusion:**

The presence of absent cochlear nerve, electrode folding, and underlying neurological disorders seemed to be associated with poorer outcomes.

## 1. Introduction

Cochlear implantation (CI) has revolutionized the life of children with hearing loss. The surgical and technological advances in CI technology have transformed the hearing rehabilitation scene in children with hearing loss. The outcomes of CI has been reported in previous literature reports to be associated with age at implantation [1], onset and duration of hearing loss [2], inner ear anatomy [3], presence of neurodevelopmental disorders [4], and level of psychosocial support [5] and rehabilitation efforts [6] following CI. The advances in preoperative imaging had allowed us to identify cochlear anomalies previously not identified, and allowed us to better predict unusual surgical anatomy and plan for optimal surgical approaches to address anticipated intraoperative challenges.

The majority of children with congenital hearing loss do not have identifiable anatomical abnormalities on imaging. Only about 20% of children have been reported to have bony cochlear malformations. Jackler's classification was coined in 1987 and was one of the most widely accepted classifications of inner ear malformations. The inner ear malformations were divided into complete labyrinthine aplasia (Michel's deformity), cochlear aplasia, cochlear hypoplasia, Mondini deformity (incomplete partition), and common cavity. [7] The first implantation in a malformed cochlea was reported by Mangabeira-Albernaz in 1983 [8]. In the 1990s, the reported literature was mostly small series. Implantation in children with inner ear malformations (IEM) was fraught with uncertainties in the clinical outcomes [9–11]. The reduced neural tissue in the inner ear makes it difficult to predict the CI responses. [12, 13]. Fortunately, with improvement in technology, various implant electrodes have been invented to facilitate the insertion of electrode and allowed maximum insertion in these cases.

Many studies have tried to compare the speech perception of patients with normal cochlear and IEM. Dettman et al. did not find a positive relationship between the degree of cochlear malformation and speech perception. [14] Eisenman et al. found that there was overall improvement in speech perception, but the progression was slower in children with IEM compared with age-matched children with normal cochlear. [15] Van Wermeskerken et al. did not find any difference in the children with osseous IEM compared with normal cochlear in close-set and open-set speech perception [16]. Papsin found that the outcomes were similar between children with normal cochlear and IEM in the speech perception, but there was an increased risk for intraoperative complications in the latter group. [3] Pakdaman et al. suggested an overall trend towards lower speech perception and an increased difficulty in surgical approaches in the group with IEM. The more severe malformations may perform worse than those with minor cochlear anomalies [17].

Comparison of the outcomes following CI surgery in children with IEM is difficult due to the heterogeneity in the patient factors and the outcomes measurement used. In this study, we would like to (a) evaluate the audition and speech perception skills before and after implantation for children with cochlear malformations and (b) to identify factors related to better and worse audition and speech perception outcomes for children with cochlear malformations who received CI.

## 2. Methods

### 2.1. Participants

The database of patients with hearing loss, between the age of 0 and 18 years, who underwent CI between the year 2000 and 2013 was retrieved from the surgical database of a single pediatric tertiary hospital in Singapore. The inclusion criteria for the study were children with bilateral profound or severe to profound sensorineural hearing loss and children with limited benefits from binaural hearing aids. The definition of profound hearing loss was defined as “hearing loss in better ear from 90 dBHL,” and severe hearing loss was defined as “hearing loss in better ear between 70–89 dBHL.” Children were considered to be receiving suboptimal benefits from the hearing aids when the aided threshold was not within the speech banana on audiogram or they had unclear speech. As part of the standard CI workup, all patients planned for CI had undergone high-resolution computed tomography (CT) scan of the temporal bone. Children who had abnormal CT scan of the temporal bone also had a magnetic resonance imaging (MRI) scan performed. All abnormal CT temporal bone scans were identified and their accompanying MRI scans reviewed by a trained neuroradiologist.

### 2.2. Materials

Children with preimplant hearing thresholds in the category of severe to profound and children with suboptimal benefits from hearing aids, with abnormal CT scans of the temporal bones or MRI were analyzed. The main outcome measure was the auditory and speech perception skills, which in this study was assessed using the Infant-Toddler Meaningful Auditory Integration Scale (IT-MAIS). The IT-MAIS, a validated and reliable auditory assessment tool, was used to assess the listening skills to measure the listening abilities in children aged 0–3 years. It comprises of 10 items grouped into 3 domains: vocalization behavior, alerting to sounds, and deriving meaning from sound. Each item is scored on a five-point scale [18, 19].

### 2.3. Methods

The data used in this study were extracted from the surgical database in the hospital using the search codes for cochlear implantation from the year 2000 to 2013. Demographic information and data on risk factors, universal newborn hearing screening (UNHS) status, type of IEM, duration of hearing aid usage, age at implantation, speech perception and audiological outcomes, and duration of follow-up were collected from clinical records of these patients. Operative details and surgical complications were recorded from the operative records. This study was approved by the institutional review board (IRB) of the hospital where the study was conducted, and ethical standards for the collection of data were met in this study. For the speech perception and audiological outcomes, the IT-MAIS was used for the younger children up to 3 years old. For children above 4 years, the aided threshold was used for audiometric assessment. The IT-MAIS was administered to the parents in a structured interview at the Otolaryngology office before the use of HA, at each review following the use of HA and prior to the CI.

## 3. Results

A total of 70 children underwent 83 CI surgeries within the study period, of whom 13 patients (18.57%) underwent bilateral CI—8 were performed simultaneously and 5 sequentially. Thirty-four (40.96%) of the CI were performed on the left side, and 49 (59.04%) were performed on the right side. There were 32 (45.71%) female patients and 38 (54.29%) male patients. The mean age of the patients was 4.05 ± 3.17 years (age range: 1–18 years) at the time of CI. The majority of patients (31 (44.29%)) underwent implantation before the age of three years, 26 children (37.14%) were implanted between three and six years of age, and 13 (18.57%) beyond six years of age (Table 1).

There were twenty patients who fulfilled the selection criteria which were stated in the methodology as children with preimplant hearing thresholds in the category of severe to profound and children with suboptimal benefits from hearing aids and with abnormal CT scans of the temporal bones or MRI (Table 2) Seventeen patients (85.00%) had profound hearing loss prior to CI, two patients (10.00%) had severe hearing loss, and one patient had severe hearing loss which progressed to profound hearing loss. The age distribution in the group with IEM was similar to the entire cohort who underwent CI, 12/20 (60.00%) were implanted before the age of three years, 5/20 (25.00%) were implanted between three and six years of age, and 3/20 (15.00%) were implanted beyond six years of age.

**Figure 1:**
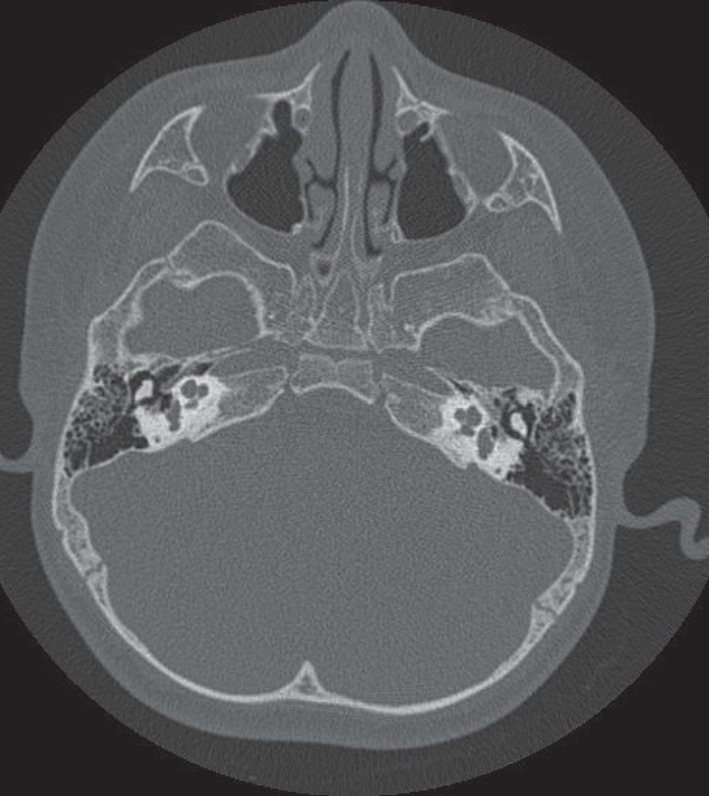
CT scan of the temporal bone with incomplete partition (IP-2).

**Figure 2:**
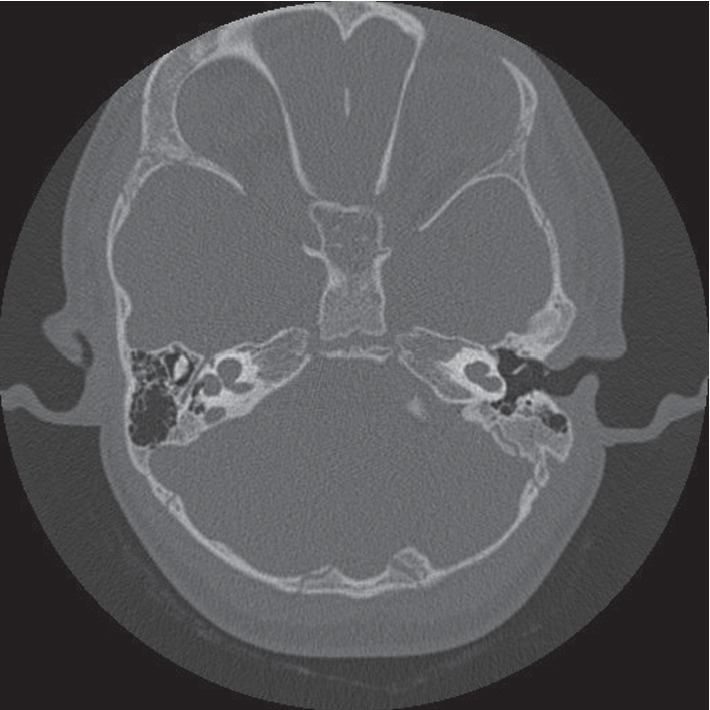
CT scan of the temporal bone with incomplete partition (IP-1).

The most common IEM seen on the CT scan was incomplete partition 2 (IP-2 type) (Figure 1), which was identified in five out of 20 patients (25.00%). Three patients (15.00%) had incomplete partition 1 (IP-1 type) of congenital IEM (Figure 2). An internal auditory canal (IAC) < 2 mm is considered narrow, and three patients (15.00%) had a narrowed (IAC) on CT scan, of which two eventually had a normal cochlear nerve (CN) visualized on MRI. Two patients (10.00%) were found to have an absent CN on MRI scan.

A facial nerve monitor (NIM-2, Xomed, Minneapolis, MN, USA) was set up for all cases with IEM. There were no incidences when the surgery had to be aborted because of anomalous facial nerve anatomy. All the cases were successfully performed using standard transmastoid facial recess approach by a single senior surgeon, and there was no facial nerve injury in this series.

The CSF gusher occurred in 15 out of 26 CI (57.69%) in the group with IEM, which was eight times higher compared to four out of 57 (7.02%) in the group with normal inner ear anatomy. These patients were managed by packing the cochleostomy with fascia. None of them had long-term complications. There were no cases of meningitis. Only one patient had lumbar drain inserted, and it was removed on postop day two without any complications (Table 1).

### 3.1. Audition and Speech Perception Outcomes

Nine out of twenty patients (45.00%) had poor IT-MAIS scores prior to implantation. The average preoperative IT-MAIS score for children with IEM was 14.1. Most of the children 8/20 (40.00%) could only imitate sounds, babble, and vocalize vowel sounds after a trial of hearing aids at a level that was not appropriate for their age. The older CI patients 3/20 (15.00%), mean age 8.33 years (range 7–10 years), were mostly referred from the Canossian School for Hearing Impaired as changeover candidates because of persistently unclear speech despite prolonged regular use of hearing aids. Most of them had aided hearing threshold that was not within the speech banana.

Of the 20 children with IEM, 11 (55.00%) had good speech and aided hearing threshold within speech limits after CI. On follow-up, these children were eligible for reintegration into mainstream schools. Five patients (25.00%) had improvement in speech but continued to receive education in the schools for hearing-impaired children. Four patients (20.00%) had poor progress after implant surgery and they remained nonverbal.

**Figure 3:**
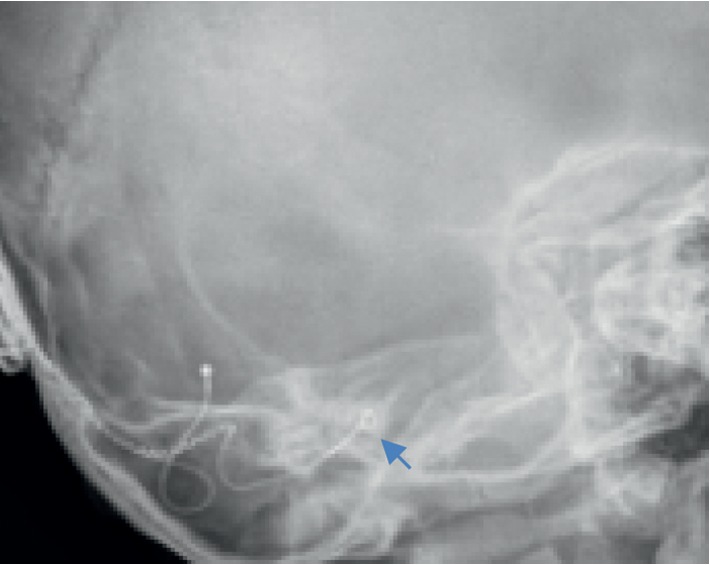
Modified Stenvers view showing folding of implant electrode.

**Figure 4:**
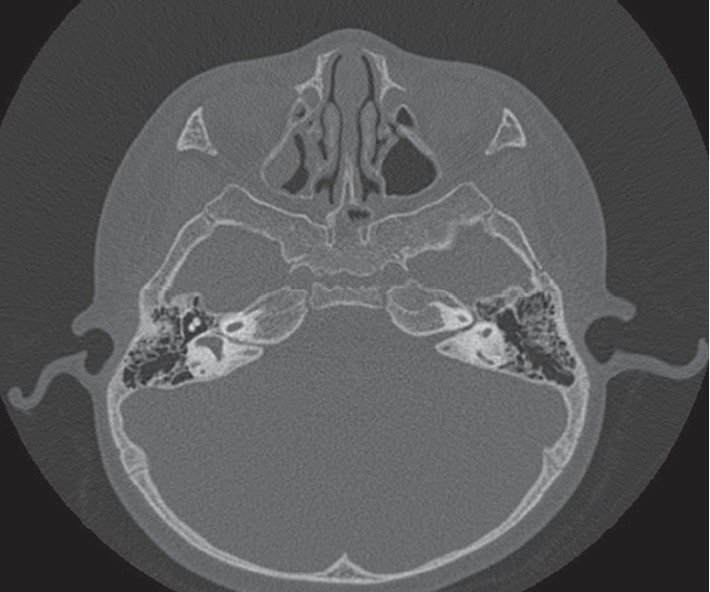
CT scan of the temporal bone with narrowed internal auditory canal (IAC).

In our study, children with absent cochlear nerve, electrode folding, and underlying neurodevelopmental disorders seemed to have poorer outcomes compared to the rest of the children with IEM who underwent CI. Isolated IEM did not result in suboptimal outcomes following CI. There were four patients with a family history of HL. Two of them remained nonverbal and two of them continued to attend the school for hearing-impaired children. The two patients who remained nonverbal had other risk factors accounting for the poor outcomes such as folded electrodes (Figure 3) and neurodevelopmental disorders. The patient with folded electrodes had CT scan findings of absent modioli, cochlear dysplasia, and incomplete partition between the apical and middle turns. He was implanted at 3 years of age. Before implant, he was on hearing aid for 8 months and was only able to produce vowel sounds. Following implant, the speech perception outcomes remained poor and aided audiogram remained below range. There were two patients with absent CN on MRI (10.00%) (Figure 4). Both of them continued to have poor speech perception following CI. Both patients failed the UNHS and was on hearing aid trial for 14 and 16 months, respectively prior to implant. Both patients were implanted before 3 years of age. Before implant both patients could only produce vowel sounds, and the IT-MAIS score was 12.5%. Following implant, both patients did not show significant improvements in the auditory and speech perception. One patient who had global developmental delay (GDD) continued to do poorly after CI. This patient had a family history of HL. He passed UNHS, and diagnosis of profound HL was made at 2 years of age. He was using HA for 5 months, and preimplant IT-MAIS was 0%. He was implanted on the right side at 2 years 9 months and the left side at 3 years of age. Following implant, he did not show significant improvements (Table 1).

## 4. Discussion

In our center, the UNHS program was started in 1998 [20]. Prior to that, hearing loss in children was often missed, resulting in delayed detection and intervention, which in turn affects the speech and language development of the child. Currently, our practice is similar to most countries. We aim to diagnose hearing loss by 3 months of age and with children fitted with hearing aids by 6 months of age. None of the children in this study with a poor outcome had delayed diagnosis in hearing loss and hearing rehabilitation.

Children with IP-EVA spectrum abnormalities frequently achieve good performances with 100% developing open-set speech perception skills, 82% using an exclusively oral communication mode, and 65% attending a mainstream school. On the other spectrum, children with severe hypoplasia may only achieve open-set speech perception skills in 50%. The communication mode will require visual supplementations in 69%, and only 50% will be attending a mainstream school [21]. From our study, isolated inner ear anomalies did not result in poorer outcomes. Majority of them, 11 (55.00%), had good speech and aided hearing threshold within speech limits after CI and were eligible for reintegration into mainstream schools.

Based on previous literature reports, age at CI was regarded as an important predictor of speech and hearing outcomes as neuroplasticity may decrease beyond three years of age, resulting in poorer outcomes [22]. None of the patients in this study with poor outcomes had CI beyond three years of age. In this study, the majority of patients 31 (44.29%) underwent implantation before the age of three years. The age distribution in the group with IEM was similar to the entire cohort who underwent CI, 12/20 (60.00%) were implanted before age of three years. Therefore, in this study, age is not the underlying cause, resulting in poor outcomes. The children who had poorer outcomes were due to the lack of cochlear nerve, technical issues resulting in folding of electrodes, and underlying neurodevelopmental disorders.

In the past, absent/hypoplastic CN was a contraindication to CI surgery. However, there has been an expanding indication in the last few years because of the useful verbal language outcomes seen in almost three quarters of them [23–25]. In our study, absent CN seems to be a predictive factor for poor outcome. Two out of the four patients with poor outcome had absent CN. Since Shelton et al. published their result on the lack of auditory response to electrical stimulation in children with a narrow IAC, there had been many reports published reporting poor or absent responses in children with absent/hypoplastic CN who had undergone CI surgery [21, 26–29]. However, on the other hand, there had been reports on children diagnosed with absent/hypoplastic CN with significant speech perception and auditory responses after CI [30–33]. The proposed theory was that the cochlear fibers could have been bundled up in the vestibular or facial nerve rather than appearing as a separate nerve on MRI [34, 35]. The other reason could be due to the limitation of the MRI resolution, resulting in a small CN that was missed [36].

At the time when the study was completed, there was no auditory brainstem implantation (ABI) in our center. Ethically, the authors felt that it was justifiable to implant the child given the low risk of complications in the hands of an experienced surgeon and the possible improvement in speech and language development in these children. There is still controversy on CI in children with absent CN. Transtympanic electrical auditory brainstem response (TTEABR) is a useful procedure that can be performed to help in the decision-making process in patients with absent/hypoplastic CN. A positive TTEABR response predicts presence of CN fibers, and the patient should proceed with the CI [28, 37, 38]. At that time when CI was performed in the two patients with absent CN, TTEABR was not available in the center. The center would now recommend using TTEABR prior to CI in patients with absent CN on MRI.

CI-evoked electrical auditory brainstem response (EABR), similar with TTEABR, can be used to assess neural integrity of the CN. Gordon et al. showed decreased latency with CI-evoked EABR with greater CI use and shorter interwave intervals with increased neural synchrony [39]. In another study by Birman et al., they found that the CI-evoked EABR was more sensitive than neural response telemetry (NRT) in eliciting a neural response [40]. As in CI-evoked EABR, electrical compound action potential (ECAP) has been shown to grow with CI stimulation. An absent ECAP response is associated with poor CI performance in children with auditory neuropathy [39, 41, 42].

The success of CI in children with inner ear malformations depends on the severity of the malformations. Most studies reported good outcomes in mild anomalies or labyrinthine anomalies with normal cochlear. Patients with more severe inner ear malformations are expected to have poorer outcomes because of the possibility of fewer spiral ganglion cells and higher risks of postoperative complications. One patient in this study had poor outcome possibly due to technical problems resulting in folded electrodes. This patient had absent modioli, cochlear dysplasia, and incomplete partition between the apical and middle turns shown on his CT scan. CSF gush was known to be higher in CI performed on an anomalous inner ear, occurring in 40 to 50% of surgeries [9, 10, 43, 44]. In this study, the CSF gush rate was 57.69%. Though most of the CSF gush could be controlled with fascia packing into the cochleostomy, the surgical complications may be increased, as in this case the anomalous cochlear and the CSF gusher could result in difficult insertion and suboptimal placement of electrodes. Besides having poor speech perception and auditory outcomes, these children are at risk of meningitis because of the communication between the anomalous cochlear and the IAC. Fortunately, none of the patients in this study developed meningitis.

Our findings were consistent with previous literature reports. The outcomes of CI in children with GDD seem to be poorer compared to the ones with normal cognitive function. One patient in this study with poor outcome had underlying neurodevelopmental disorders. His poor outcome could be due to other central problems resulting in slower development in general, including the speech domain. A child with underlying neurological abnormalities may have a poorer outcome after CI, and careful counselling is necessary to manage parental expectations [27, 45].

Aberrant facial nerves were reported in 16% of inner ear malformations and even more frequently in patients with severe malformations [46]. None of the patients in our study had a facial nerve injury. The facial nerve monitor was not routinely setup in our center if the patient had a normal anatomy, but use of a nerve monitor in this group of patients is strongly recommended due to the high incidences of abnormalities.

The limitations of our study were the lack of a standardized reporting format of the audiological and speech outcome before and after CI and the lack of long-term follow-up data. Most of the clinical information was written in a descriptive manner. Long-term outcome was not available in our clinical charts for some patients who were followed up by the audiologist in the special school. The relatively small patient number included in this study and having no control group to compare the outcomes were also limitations we acknowledged. A prospective study studying the factors affecting the CI outcomes would be recommended.

## 5. Conclusion

Children with poorer outcomes following CI are unlikely due to having isolated inner ear malformations (IEM). Based on this study, the contributing factors could be due to absent CN and background GDD in addition to having IEM. However, performing CI in children with IEM was technically more challenging because of the abnormal anatomy, possible anomalous facial nerve course, and higher incidence of CSF gush. The decision to implant in patients with absent CN remains a debatable issue.

## Figures and Tables

**Table 1:** Demographics and clinical characteristics of patients undergoing CI.

Demographics and clinical characteristics of patients undergoing CI	
Age at CI (years)	
Mean	4.05 ± 3.17 years (range 1–18)
<3	31 (44.29%)
3–6	26 (37.14%)
>6	13 (18.57%)
Gender	
Male	38 (54.29%)
Female	32 (45.71%)
Abnormal CT scan	20 (28.57%)
Complications	
CSF gush	15 (57.69%)
Facial nerve injury	0 (0%)
Speech and hearing before CI	
Poor IT-MAIS score	9/20 (45.00%)
Poor speech (imitate sounds, or babbles)	8/20 (40.00%)
Unclear speech	3/20 (15.00%)
Speech and hearing after CI	
Reintegration into mainstream	11/20 (55.00%)
School for special needs children	5/20 (25.00%)
Poor response	4/20 (20.00%)

CI: cochlear implant; CSF: cerebrospinal fluid; CT scan: computed tomography scan; IT- MAIS: Infant-Toddler Meaningful Auditory Integration Scale.

**Table 2:** Table summarizing age at implant, inner ear anomalies, intraop complications, and outcome.

Sex	Age at implant	CT malformation	Intraoperative complications	Additional medical issues	Type of implant	Outcome
F	R: 1 yr 7 months	R: IP-1	R: none	None	R: CI 24 Re	Good. Speaking sentences
	L: 2 yrs 9 months	L: IP-1	L: CSF gush		L: CI 24 Re	

M	R: 3 yrs 6 months	IP-1	CSF gush	None	Med-el Sonata	Good. Attends primary school mainstream

M	R: 10-yr-old	EVA and modiolus dysplasia	None	None	Nucleus Freedom	Attends school for hearing impaired

F	R and L both at 1 year 1 month	R: IP-2	R: CSF gush	Gentamicin use	R and L: Med-el Sonata	Good. Attends primary school mainstream
		L: IP-2	L: none			

F	R: 3 yrs 8 months	R: mild dilated vestibule and posterior SCC	None	Had implant on the left side which is normal side on CT at 1-yr-old	R: CI 24 Re	Good. Can speak in sentences
					L: CI 512	

M	R: 1 yr 11 months	R: IP-1	CSF gush (mild)	NNJ with phototherapy	R: CI 24 Re	Poor
		MRI: absent CN				

M	R: 3-yr-old	CT: bilateral absent modioli, slight dysplasia of the cochleas, mild incomplete partition between apical and middle turns	CSF gush (massive)	Folded electrodes on X-ray	R: CI 24 Re	Poor

F	L: 2 yrs 8 months	CT: bilateral narrow IAC and CN apertures. CN hypoplasia or agenesis	CSF gush (mild)		L: CI 512	Poor
		MRI: bilateral IAM narrowed. Worse on the right side. Absent right vestibulocochlear nerve, left CN is likely absent as well				

M	R: 2 yrs 9 months	IP-2 and EVA	CSF gush		R: CI 512	Good speech but articulation still can be improved

F	L: 1 yr 9 months	CT: left CN canal at lower limit calibre, right normal	None		L: Med-el Sonata	Good
		MRI: CN are normal bilaterally				

M	R: 2 yrs 9 months	CT: narrowed CN canals, CN abnormalities	None	Dad has hearing loss GDD	R and L: CI 512	Poor progress, not able to produce formed words 1 year after bilateral CI
	L: 3 yrs	MRI: normal CN				

F	L: 7 yrs 5 months	CT: IP-2 and EVA	CSF gush (mild)	Both parents are hearing impaired and mute	L: CI 512	Followed up in school for hearing impaired

F	R: 2 yrs	CT: bilateral fenestral otosclerosis which may be related to congenital rubella infection	None	Congenital rubella infection	R: CI 24 Re (done in USA)	Good speech
	L: 4 yrs 7 months				L: CI 512	

M	R: 3 yrs	CT: IP-2 and EVA	CSF gush (mild)		R: CI 512	Aided threshold within range

F	R: 2 yrs 7 months	CT: cochlear dysplasia and dilated vestibule bilaterally	CSF gush (mild)	Johnson–Blizzard syndrome. Hypothyroidism. Lumbar drain inserted	R: CI 24 Re	Good progress. Aided threshold within range. Trying for mainstream

F	R: 3 yrs	CT: mondini variant dysplastic modiolus and EVA	CSF gush (moderate)	Mild NNJ: both parents have hearing loss	R: CI 24 Re	Attends school for hearing impaired

M	R: 8 yrs	CT: EVA with incomplete partition between middle and apical cochlear turns. IP-2. Bilateral.	CSF gush (mild)		R: CI 24 Re	Fair speech. Attends school for hearing impaired.

M	L: 3 yrs 2 months	CT: bilateral basal turns of cochlear dilated, incomplete septation of middle and apical turns, and absent modiolus. No EVA. Bilateral Inner ear dysplasia.	CSF gush		L: Sonata Ti 100 and standard electrode	Poor response. Aided threshold not within range
		MRI: bilateral Inner ear dysplasia. Hypoplastic left CN. Right CN vaguely seen and is even smaller.				

F	L: 4 yrs 5 months	CT: right prominent vestibular aqueduct	None		L: CI 24 Re	Aided threshold within range

M	R and L: 1 year 4 months	CT: bilateral short and dilated posterior SCC.	None		R and L: CI 24 Re	Aided threshold within range

IP-1: incomplete partition 1; IP-2: incomplete partition 2; EVA: enlarged vestibular aqueduct; CSF: cerebrospinal fluid; SCC: semicircular canal; CN: cochlear nerve; NNJ: neonatal jaundice; IAC: internal auditory canal; IAM: internal auditory meatus; GDD: global developmental delay.

## Data Availability

The datasets generated during and/or analysed during the current study are available from the corresponding author on reasonable request.
